# Concentration of Selected Adipokines and Factors Regulating Carbohydrate Metabolism in Patients with Head and Neck Cancer in Respect to Their Body Mass Index

**DOI:** 10.3390/ijms24043283

**Published:** 2023-02-07

**Authors:** Jarosław Nuszkiewicz, Jolanta Czuczejko, Wiktor Dróżdż, Alina Woźniak, Bogdan Małkowski, Karolina Szewczyk-Golec

**Affiliations:** 1 Department of Medical Biology and Biochemistry, Faculty of Medicine, Ludwik Rydygier Collegium Medicum in Bydgoszcz, Nicolaus Copernicus University in Toruń, 24 Karłowicza St., 85-092 Bydgoszcz, Poland; 2 Department of Psychiatry, Faculty of Medicine, Ludwik Rydygier Collegium Medicum in Bydgoszcz, Nicolaus Copernicus University in Toruń, 9 M. Curie Skłodowskiej St., 85-094 Bydgoszcz, Poland; 3 Department of Nuclear Medicine, Oncology Centre Prof. Franciszek Łukaszczyk Memorial Hospital, Bydgoszcz, 2 dr I. Romanowskiej St., 85-796 Bydgoszcz, Poland; 4 Department of Diagnostic Imaging, Faculty of Health Sciences, Ludwik Rydygier Collegium Medicum in Bydgoszcz, Nicolaus Copernicus University in Toruń, 2 dr I. Romanowskiej St., 85-796 Bydgoszcz, Poland

**Keywords:** adipokines, biomarkers, body mass index, carbohydrate metabolism, cytokines, head and neck cancer, obesity

## Abstract

Head and neck cancers (HNCs) are a group of tumors not common in European populations. So far, not much is known about the role of obesity, adipokines, glucose metabolism, and inflammation in the pathogenesis of HNC. The aim of the study was to determine the concentrations of ghrelin, omentin-1, adipsin, adiponectin, leptin, resistin, visfatin, glucagon, insulin, C-peptide, glucagon-like peptide-1 (GLP-1), plasminogen activator inhibitor-1 (PAI-1), and gastric inhibitory peptide (GIP) in the blood serum of HNC patients depending on their body mass index (BMI). The study included 46 patients divided into two groups according to their BMI values: the normal BMI group (nBMI) included 23 patients with BMI < 25 kg/m^2^ and the increased BMI group (iBMI) included patients with BMI ≥ 25 kg/m^2^. A control group (CG) included 23 healthy people (BMI < 25 kg/m^2^). Statistically significant differences in the levels of adipsin, ghrelin, glucagon, PAI-1, and visfatin were shown between nBMI and CG. In the case of nBMI and iBMI, statistically significant differences were observed in the concentrations of adiponectin, C-peptide, ghrelin, GLP-1, insulin, leptin, omentin-1, PAI-1, resistin, and visfatin. The obtained results indicate a disruption of endocrine function of adipose tissue and impaired glucose metabolism in HNC. Obesity, which is not a typical risk factor for HNC, may aggravate the negative metabolic changes associated with this type of neoplasm. Ghrelin, visfatin, PAI-1, adipsin, and glucagon might be related to head and neck carcinogenesis. They seem to be promising directions for further research.

## 1. Introduction

Head and neck cancers (HNCs) are a significant clinical and social problem. It is estimated that over 500,000 patients are diagnosed with HNC each year worldwide [1]. In 2020, there were 151,000 new cases of HNCs in Europe [2]. HNCs are a rare group of neoplasms characterized by an unfavorable prognosis, despite the significant development of diagnostic and therapeutic methods [3]. HNCs affect the organs of the head and neck, such as the lip, oral cavity, tongue, gum, pharynx, tonsil, larynx, paranasal sinuses, and salivary glands [4]. The neoplasms in this group are quite homogeneous in terms of morphology. The vast majority of HNCs include squamous cell carcinomas of varying degrees of differentiation, originating from the epithelium of the mucous membranes [5]. So far, several risk factors favoring the development of HNC have been identified. Irritation of the mucous membranes of the oral cavity and throat with cigarette smoke or alcohol, as well as chronic, mechanical damage to the tissues through ill-fitting dentures or broken teeth, may lead to carcinogenesis [6]. *Human papillomavirus* (HPV) and Epstein-Barr virus (EBV) infections may also be a cause of HNC [5,7,8]. The South Asian population is particularly vulnerable to HNCs, which may be related to socio-economic conditions [9]. The symptoms of HNC vary depending on the initial location of the cancer. Typical symptoms include pain and ulceration of the affected tissue, which over time may lead to breathing, swallowing, and speech difficulties [10]. Some patients with HNC have enlarged lymph nodes in the neck [4]. Surgery, chemotherapy, and radiotherapy are the most common forms of treatment for HNC patients. These methods are usually used together in combination therapy [11].

Obesity is a chronic disease of complex etiology associated with abnormal or excessive accumulation of adipose tissue, which poses a health risk [12]. It is estimated that 2.1 billion people in the world may be overweight or obese [13]. One of the methods of diagnosing obesity is by measuring the patient’s body mass and height to calculate the body mass index (BMI) [13]. In people with a BMI ≥ 25 kg/m^2^, a greater amount of adipose tissue may be accumulated [14]. Adipose tissue not only stores excess energy but also produces various types of substances [15]. Adipokines, hormones secreted by white adipose tissue, regulate inflammatory and metabolic processes as well as influence cell growth and proliferation [16]. Adipokines make obesity inextricably linked to low-grade chronic inflammation [17]. Inflammatory processes increase the risk of developing cancer [18]. According to a meta-analysis by Dobbins et al. [19], obesity is associated with a higher risk of colon, renal, gallbladder, pancreatic, leukemia, and breast cancer. In the course of cancer, metabolic pathways of carbohydrates and lipids are disturbed [20]. Although obesity is not a typical risk factor for HNC, the coexistence of obesity and HNC may have a significant impact on the course and prognosis of the disease [21]. The Cho et al. [22] study showed that being underweight (BMI < 18.5 kg/m^2^) leads to a higher mortality in HNC patients, while obesity is associated with a better prognosis and patient survival. White adipose tissue inflammation in obesity may lead to insulin resistance and lower disease-free survival rates in HNC patients [21]. The level of adipose tissue appears to influence the course and prognosis of HNC. Thus, the ambiguous role of obesity and its complications as predisposing factors for HNC requires further research. 

So far, only a few studies concentrated on the role of adipokines and glucose metabolism in patients with HNC have been conducted. Therefore, the aim of the presented study was an attempt to find potential links between obesity and HNC and to identify analytes related to obesity and glucose metabolism that may be associated with HNC carcinogenesis. For this purpose, it was planned to determine the concentrations of ghrelin, omentin-1, adipsin, adiponectin, leptin, resistin, visfatin, glucagon, insulin, C-peptide, glucagon-like peptide-1 (GLP-1), plasminogen activator inhibitor-1 (PAI-1), and gastric inhibitory peptide (GIP) in the course of HNC. Including HNC patients with both BMI values in the reference range and with elevated BMI values in the study could allow for a better understanding of the role of selected adipokines and factors regulating glucose metabolism in the course of HNC. 

## 2. Results

The results of the anthropometric analyzes and clinical characteristics of the study participants are presented in Table 1. The group of patients with HNC was divided into two subgroups depending on BMI. The study included 23 HNC patients with normal body mass index values (nBMI) and 23 HNC patients with increased BMI (iBMI). The third group participating in the study included 23 healthy volunteers who qualified as a control group. There was a statistically significant difference in the body mass and BMI between the nBMI and iBMI groups. No significant differences were found between the nBMI and control groups in anthropometric and clinical characteristics.

In the nBMI patients, statistically higher levels of adiponectin, omentin-1, and ghrelin were observed compared to the participants from the iBMI group. Individuals from the iBMI group presented significantly higher concentrations of insulin, leptin, C-peptide, GLP-1, PAI-1, resistin, and visfatin than the patients from the nBMI group. No significant differences were found between the nBMI and iBMI groups in the case of adipsin, GIP, and glucagon. The results of biochemical analyzes performed in HNC patients from the nBMI and iBMI groups are presented in Table 2 and Table 3.

In the nBMI group, statistically significant higher levels of adipsin, visfatin, glucagon, and PAI-1 were observed compared to the control group. Moreover, a statistically lower level of ghrelin was noted in the nBMI group compared to the control group. The analysis of the results showed no differences in the levels of omentin-1, GIP, adiponectin, C-peptide, GLP-1, insulin, leptin, and resistin between the nBMI and control groups. Table 4 and Table 5 present the results of a laboratory analysis for the nBMI patients and the control group.

**Figure 1:**
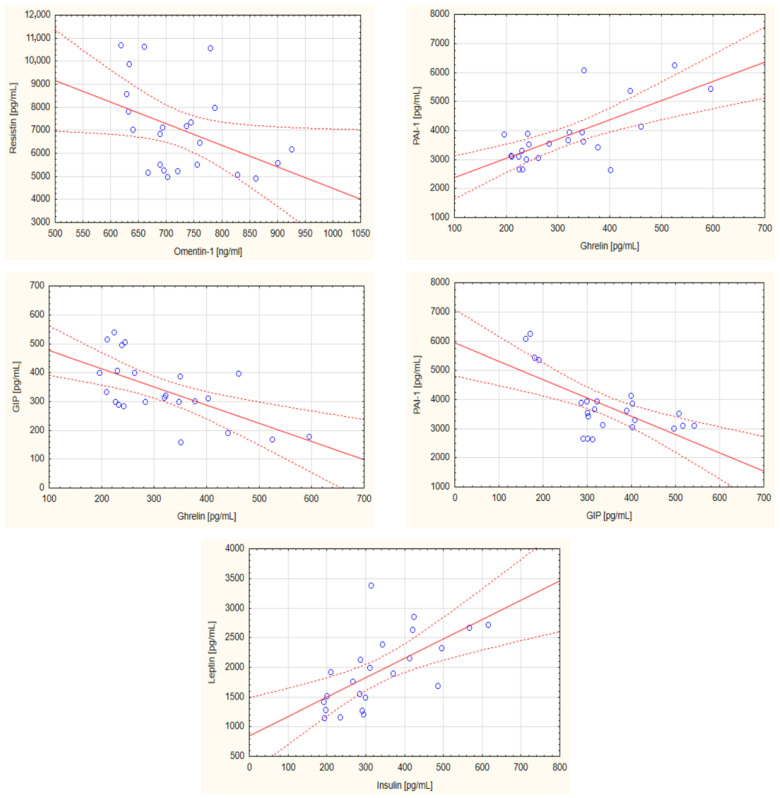
Statistically significant correlations between selected biochemical parameters in the normal body mass index group of head and neck cancer patients. Abbreviations used: GIP: glucose-dependent insulinotropic polypeptide; PAI-1: plasminogen activator inhibitor-1. The regression line is marked with a solid line, while the confidence intervals of 0.95 are marked with a dashed line. *p* < 0.05 was considered as statistically significant.

**Figure 2:**
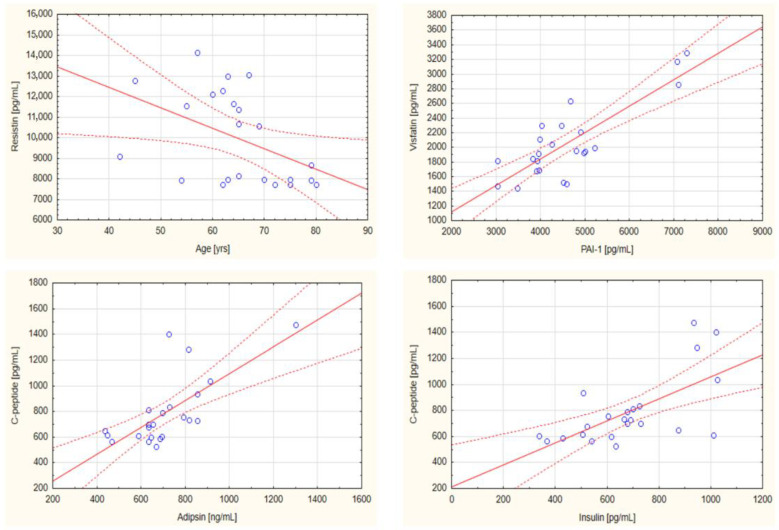
Statistically significant correlations between selected biochemical parameters in the increased body mass index group of head and neck cancer patients. Abbreviations used: PAI-1: plasminogen activator inhibitor-1. The regression line is marked with a solid line, while the confidence intervals of 0.95 are marked with a dashed line. *p* < 0.05 was considered as statistically significant.

The correlation between the concentrations of the analyzed biomarkers was also evaluated. In the nBMI group, statistically significant negative correlations were observed between ghrelin and GIP (r = −0.6284; *p* = 0.001), GIP and PAI-1 (r = −0.6662; *p* = 0.001), and omentin-1 and resistin (r = −0.4233; *p* = 0.044), whereas a significant positive correlation was found between ghrelin and PAI-1 (r = 0.6964; *p* < 0.001) as well as between insulin and leptin (r = 0.6359; *p* = 0.001). Figure 1 presents the significant correlations between selected parameters in the nBMI group. In the iBMI group, a negative correlation between age and resistin (r = −0.4479, *p* = 0.032) and a positive correlation between PAI-1 and visfatin (r = 0.8253; *p* < 0.001), adipsin and C-peptide (r = 0.7054; *p* < 0.001), as well as between insulin and C-peptide (r = 0.6462; *p* = 0.001) were noted. The statistically significant correlations in the iBMI group are presented in Figure 2.

Analogous statistical analysis was performed separately for the groups of women (nBMI vs. iBMI and nBMI vs. control group) and men (nBMI vs. iBMI and nBMI vs. control group). However, no statistically significant gender-specific differences or correlations were found. This could be due to the small number of subjects in the groups, which were additionally divided by gender. Nevertheless, although men are more likely to develop and die from HNC due to their smoking and hard alcohol consumption habits, gender is not a typical risk factor for HNC, which may have been the reason why we did not observe gender-specific differences in the studied parameters.

## 3. Discussion

In the course of both obesity and carcinogenesis, changes in the metabolic profile of adipose tissue have been observed [23,24,25]. This can lead to increased synthesis and secretion of many bioactive compounds, such as hormones, adipokines, inflammatory cytokines, and growth factors [26,27,28]. To date, more than 600 adipokines have been discovered. Most of the described adipokines play a key role in maintaining carbohydrate-lipid homeostasis [29]. These factors secreted by white adipose tissue contribute to the initiation and progression of several types of cancer by stimulating the metabolic reprogramming of cells [30]. Adipokines affect tumor metabolism and lead to cancer cell growth, proliferation, migration, invasion, epithelial-mesenchymal transition, angiogenesis, metastasis, and the development of multidrug resistance [31,32,33,34]. Low-grade chronic inflammation associated with obesity shapes the tumor microenvironment, affecting cell plasticity through epithelial-mesenchymal transition, dedifferentiation, immune cell polarization, reactive oxygen species, cytokines, and epigenetic mechanisms [35,36]. Liver, bladder, lung, colorectal, and gastric cancers are strongly associated with chronic inflammation [37,38]. Numerous studies have focused on the role of inflammation in carcinogenesis and the pharmacological reduction of inflammation as a potential anti-cancer therapy [35,39,40]. Cancer cells, due to their rapid proliferation, are characterized by a significant demand for glucose [20,41]. Increased glucose metabolism in neoplastic cells depends, inter alia, on their localization, increased expression of glucose transporting proteins from the glucose transporter (GLUT) family, enzymes such as phosphoglucomutase or hexokinase, the degree of cell proliferation, and the tumor vascularity [42]. HNCs have not been the subject of numerous studies, especially in the aspect of adipokine homeostasis and factors regulating carbohydrate-lipid metabolism. Therefore, the presented study might be a valuable contribution to the current state of knowledge in this area.

Ghrelin, also called “hunger hormone”, is a short polypeptide hormone synthesized by enteroendocrine cells of the gastrointestinal tract [43]. Ghrelin is a hormone that regulates food intake and is secreted during fasting [43]. In people with obesity, a decrease in ghrelin levels is often observed [43]. In the course of HNC, we observed that ghrelin levels were lower in the iBMI patients compared to the nBMI patients. In the healthy individuals, we observed a higher level of ghrelin than in the nBMI patients. These results may suggest improper secretion of ghrelin in HNC patients and/or a relationship between this hormone and HNC carcinogenesis. It should be emphasized that patients with HNC have problems with food intake, which may lead to food restriction and starvation. According to Stempniewicz et al. [44], ghrelin presents anti-inflammatory, antioxidative, and antiapoptotic effects in oral mucositis. Hiura et al. [45] observed that during anti-cancer cisplatin-based chemotherapy, ghrelin levels were reduced in patients with advanced esophageal cancer.

Omentin-1, also known as intelectin 1, is a 34-kDa adipokine secreted primarily by visceral white adipose tissue [46]. It belongs to the group of adipokines with anti-inflammatory and antioxidant properties [46]. Recent studies have indicated that omentin-1 promotes insulin-dependent glucose transport in adipocytes [47]. The level of omentin-1 negatively correlates with BMI, especially in the course of type 2 diabetes mellitus [48]. In the presented study, the level of omentin-1 was significantly higher in the nBMI group compared to the iBMI group. No difference in serum omentin-1 concentration was observed between the HNC patients with normal BMI and the healthy controls. The obtained results could be explained by the disturbed hormonal activity of increased adipose tissue in HNC patients with increased BMI rather than by the influence of the neoplasm. Disturbed secretion of omentin-1 has been found in some studies concerning other cancer types. Shen et al. [49] described a study on a group of 41 patients with renal cell carcinoma. The authors indicated a significantly lower level of omentin-1 in the cancer patients compared to the control group. Both groups were characterized by a mean BMI in the reference range. This study did not include obese patients; however, researchers indicated a negative correlation between BMI and omentin-1 concentration. The role of selected adipokines in the course of postmenopausal breast cancer was described by Christodoulatos et al. [50]. The study group consisted of 103 females with a mean BMI of 27.7 ± 4.14 kg/m^2^. The mean level of omentin-1 in the serum was significantly lower in the case of cancer patients compared to the control group. The authors indicated that the omentin-1 level was inversely correlated to the metabolic and inflammatory biomarkers, the cancer stage, and the number of infiltrated lymph nodes. Researchers suggested that the concentration of circulating omentin-1 may be a diagnostic marker for breast cancer. Currently, the mechanisms linking omentin-1 to tumor development remain unknown, but studies have indicated that omentin-1 affects the course of cancers with different localizations [51].

Adipsin, also called complement factor D, is a serine protease secreted mainly by adipocytes. It functions as an activator of the alternative pathway of the complement system [52]. Increased BMI and a greater volume of adipose tissue are associated with a high level of adipsin [53]. In the presented study, there were no differences in adipsin levels between the HNC groups. A statistically significant lower adipsin concentration was observed in the healthy people in the control group compared to the nBMI group. These results suggest that adipsin might be a factor associated with carcinogenesis. This is in accordance with the results of other studies. In the study of Nezhad et al. [54], the cell lines UT-SCC-12A, UT-SCC-91, UT-SCC-105, UT-SCC-111, and UT-SCC-118 were used as models of cutaneous squamous cell carcinoma. It was observed that adipsin increased the proliferation of each cell line cell through the regulation of the extracellular signal-regulated kinases 1/2 (ERK1/2) signaling pathway. Mizuno et al. [55] indicated that adipsin and its downstream effector, hepatocyte growth factor, are active players in adipocyte-cancer cell interactions.

Adiponectin is a polypeptide hormone produced and secreted by adipocytes [56]. In the course of obesity and hypotrophy of adipose tissue, a decrease in the level of adiponectin is observed [57]. Adiponectin affects a number of metabolic processes, especially the metabolism of glucose and fatty acids in the liver and muscles, indirectly affecting the sensitivity of tissues to insulin and reducing inflammation [21,57]. Decreased adiponectin levels may lead to carcinogenesis [56]. Our results indicate that the obese HNC patients presented lower adiponectin levels compared to the nBMI group. No difference in adiponectin concentration was observed between the HNC patients with a normal BMI and the healthy individuals. Howard et al. [58] indicated that low adiponectin receptor 1 expression may be a predictor of improved overall survival in patients with oesophageal cancer.

Leptin is a well-known adipokine described as a pro-inflammatory biomolecule synthesized and secreted by white adipose tissue. It acts by activating transmembrane receptors (Ob-Rs) [59]. A positive correlation between the concentration of leptin in the blood plasma and the patient’s BMI or the volume of adipose tissues has been found in numerous studies [59]. In our study, we observed higher leptin levels in the iBMI patients than in the nBMI group, as well as no differences between the nBMI group and the control group. In the article by Ozsoy et al. [60], male patients with HNC were tested for leptin levels. A group of pre-treatment (*n* = 40) patients with a BMI of 24.96 ± 4.02 kg/m^2^ was compared with a control group with a BMI of 28.85 ± 3.52 kg/m^2^. The results of the study indicated that the concentrations of leptin in the pre-treatment and control groups were similar. A statistically significant lower level of leptin was shown only in the group of patients with HNC post-treatment (*n* = 40; BMI 23.08 ± 3.92 kg/m^2^) compared to the control group. Leptin can bind to Ob-R on breast cancer cells and enhance several tumor cell responses in tumor tissue by inappropriately activating multiple signaling pathways, such as MAPK and ERK1/2 activation, signal transducer and activator of transcription 3 (STAT3), and phosphatidylinositol 3-kinase/protein kinase B (PI3K/Act) [61].

Resistin is a pro-inflammatory adipokine [62]. This polypeptide triggers cellular insulin resistance, stimulates the endothelium to accumulate lipids, and exhibits immunomodulatory properties [62]. Obesity and metabolic syndrome are associated with an elevated level of resistin [62]. In neoplastic diseases, resistin may enhance neoangiogenesis and metastasis by modulating vascular endothelial growth factor (VEGF) secretion [62]. In our study, the HNC patients with an increased BMI presented higher levels of resistin compared to the nBMI group. No differences were observed between the nBMI patients and the healthy control group. Nakajima et al. [63] studied resistin levels in patients with squamous cell carcinoma of the esophagus. The patients with a more advanced stage of the disease showed higher concentrations of resistin. Thus, it was suggested that resistin could be a potential biomarker for squamous cell carcinoma of the esophagus.

Visfatin, also known as nicotinamide phosphoribosyltransferase, is an enzyme belonging to the group of adipokines [64]. Visfatin is a rate-limiting enzyme in the biosynthesis of nicotinamide adenine dinucleotide (NAD^+^) [65]. This adipokine is a pro-inflammatory protein, and its level increases in obesity, insulin resistance, and cardiometabolic diseases [66]. Recent studies have indicated that visfatin is involved in adipose tissue fibrosis [66]. In the course of breast cancer, the level of serum visfatin is elevated, and as a result, the promotion of the G1-to-S phase transition of the cell cycle is observed [65]. Additionally, visfatin leads to the activation of the nuclear factor kappa-light-chain-enhancer of activated B cells/neurogenic locus notch homolog protein 1 (NF-κB/Notch1), Act/extracellular signal-regulated kinases 1/2 (ERK1/2), and silent mating type information regulation 2 homolog 1 (SIRT1)/acetylated p53 signaling pathways [65]. In our study, the HNC patients had higher levels of visfatin than the healthy individuals. Moreover, the nBMI group presented a lower visfatin concentration than the iBMI group. Undoubtedly, these results point to the influence of visfatin in HNC carcinogenesis. Abdulsalam et al. [67] reported that visfatin levels were significantly higher in colon cancerous tissue compared to the paired adjacent non-cancerous tissue.

Glucagon is a hormone consisting of 29 amino acids, produced by the alpha cells of the islets of the pancreas [68]. Obesity can lead to impaired postprandial glucagon secretion [69]. What is more important is that glucagon not only regulates glucose balance but also energy homeostasis [69]. In this study, no differences in glucagon levels were observed between the nBMI and iBMI groups. On the other hand, a significantly lower concentration of glucagon in the blood serum of healthy people was observed compared to the nBMI group. These results may indicate a more intensive glucose metabolism in patients with HNC. According to Yagi et al. [70], glucagon may promote colon cancer cell growth by regulating the 5′ adenosine monophosphate-activated protein kinase (AMPK) and mitogen-activated protein kinase (MAPK) pathways.

Insulin is an anabolic peptide hormone with glucagon-antagonistic activity. Higher insulin levels are usually associated with obesity [71]. This phenomenon is related to the role of insulin, namely the intensification of lipid deposition in adipose tissue [71]. Obesity may lead to insulin resistance and even to the development of type 2 diabetes mellitus [71]. High insulin levels in hyperinsulinemia activate insulin/IGF signaling pathways followed by the activation of (PI3K)/Akt/mammalian rapamycin (mTOR) and MAPK signaling pathways, thus promoting cancer cell growth, survival, and mobility [72,73]. In our study, we observed significantly higher insulin levels in the iBMI patients compared to the nBMI patients. No difference in the serum insulin concentration was observed between the HNC patients with a normal BMI and the healthy control group. Vilaseca et al. [74] indicated that in patients with head and neck squamous cell carcinomas, glycemic metabolism is altered, which results in dysregulation of the insulin-glucagon system.

C-peptide is a metabolically inactive short peptide produced in the beta cells of the pancreas during the conversion of proinsulin to insulin [75]. In our study, the HNC patients with a normal body mass presented lower concentrations of C-peptide in their blood serum compared to the iBMI group. No difference in the serum C-peptide level was observed between the nBMI patients and the healthy controls. The meta-analysis by Guo et al. [76] indicated that the C-peptide concentration was not associated with an increased risk of prostate cancer. On the other hand, the studies of Arcidiacono et al. [77] indicated that the level of C-peptide may correlate with the development of Barrett’s esophageal carcinogenesis. The role of C-peptide in the course of neoplastic diseases remains ambiguous. According to Thota et al. [78], obesity remains a factor influencing the increase in serum C-peptide concentration.

GLP-1 is a hormone secreted by the enteroendocrine cells of the gastric mucosa in response to food intake [79]. The effect of GLP-1 on tissues has been found to be impaired in obesity, which might be related to reduced GLP-1 secretion and/or reduced insulinotropic potency [80]. In our study, we observed that the average GLP-1 level was higher in the iBMI group than in the nBMI group. There were no differences between the nBMI group and the control group. Thus, the difference observed between the groups of HNC patients could be associated with the improper regulation of GLP-1 secretion in obesity. As in the case of GIP, the role of GLP-1 in carcinogenesis is not fully understood. A few studies have indicated that GLP-1 may be associated with pancreatic cancer [81].

PAI-1, or serpin E1, is a single-chain glycoprotein that belongs to the family of serine protease inhibitors [82]. PAI-1 has been considered to have pro-cancer and pro-inflammatory properties [83,84]. In the presented study, the level of PAI-1 was lower in the healthy controls than in the HNC patients with normal BMI. In addition, the iBMI group presented a higher concentration of PAI-1 than the nBMI group. These results could strongly suggest the involvement of PAI-1 in HNC carcinogenesis. In the study by Pavón et al. [85], a high expression of PAI-1 in HNC patients was shown to increase the risk of metastatic recurrences after therapy due to an increase in tumor cell migration and resistance to cisplatin. The authors also indicated that a higher expression of PAI-1 was associated with a poor prognosis. PAI-1 might lead to PI3K/Act pathway activation. PAI-1 is involved not only in tumor metastasis but also in angiogenesis and has been found to accelerate the growth of tumor cells [83].

GIP is a short peptide produced by the mucosa of the small intestine, and its main action is to stimulate glucose-dependent insulin secretion by pancreatic β cells [86]. In the course of obesity, GIP synthesis and secretion are dysregulated [81]. In our study, we did not observe differences in the GIP levels in the patients participating in the experiment. This may be due to the very short biological half-life of GIP [81]. However, it has been found that incretins, which include GIP, might be related to pancreatic cancer [81].

**Figure 3:**
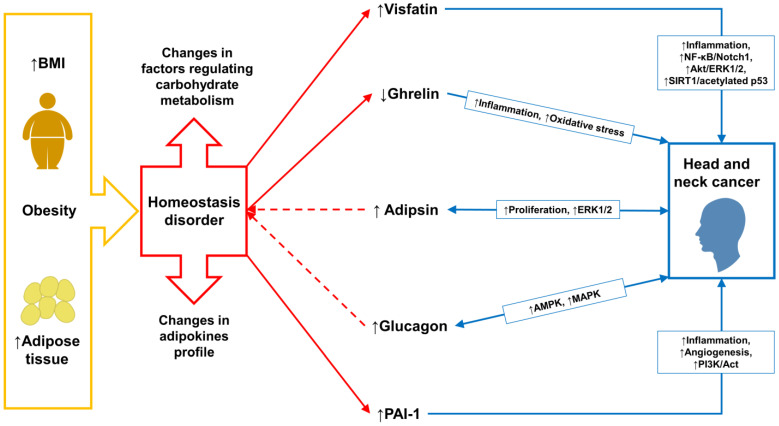
Possible interactions between impaired adipose tissue function in obesity and head and neck cancer. Abbreviations used: Act/ERK1/2: protein kinase B/extracellular signal-regulated kinases 1/2; AMPK: 5′ adenosine monophosphate-activated protein kinase; BMI: body mass index; MAPK: mitogen-activated protein kinase; NF-κB/Notch1: nuclear factor kappa-light-chain-enhancer of activated B cells/neurogenic locus notch homolog protein 1; PAI-1: plasminogen activator inhibitor-1; PI3K/Act: phosphatidylinositol 3-kinase/protein kinase B: SIRT1: silent mating type information regulation 2 homolog 1; dotted arrows—putative interactions.

The differences in omentin-1, adiponectin, leptin, resistin, insulin, C-peptide, and GLP-1 levels observed between the nBMI and iBMI groups in this study may be related to the differences in BMI and the volume of white adipose tissue. According to the results of the presented study, the influence of the above-mentioned parameters on HNC carcinogenesis seems questionable. However, statistical analysis revealed significant differences in adipsin and glucagon levels between the nBMI group and the healthy control. Thus, these parameters may be associated with HNC carcinogenesis. In turn, average serum concentrations of visfatin and PAI-1 were the highest in the iBMI group and the lowest in the control group. The opposite relationship was observed in the case of ghrelin. The contribution of those biomarkers to the course of HNC and obesity remains ambiguous. Ghrelin, visfatin, and PAI-1 may participate in HNC carcinogenesis and accompany obesity. Figure 3 presents potential interactions between obesity, adipokines, and HNCs.

The presented study has some limitations, including the small number of participants. However, to the best of the authors’ knowledge, no study has been conducted with the participation of patients with HNC from the European population in which a wide range of parameters related to the endocrine role of adipose tissue, homeostasis of carbohydrate metabolism, and inflammation were simultaneously analyzed.

## 4. Materials and Methods

### 4.1. Study Subjects

This study involved 46 patients diagnosed with HNC. The condition for the inclusion of the patient in the study included the diagnosis of malignant neoplasms of the lip, oral cavity, or pharynx (according to the International Classification of Diseases—11th Revision (ICD-11): 2B60–2B69, 2B6A–2B6D), malignant neoplasms of the larynx (according to ICD-11: 2C23), or *carcinoma* in situ of the lip, oral cavity, or pharynx (according to ICD-11: 2E60.0) [87]. Exclusion criteria included the presence of acute and chronic diseases (infectious, autoimmune, genetic, and inflammatory) other than HNC and obesity. The assessment was made on the basis of a medical interview with the patient and an analysis of the patient’s medical records. The study participants were divided into two groups of 23 patients each based on their BMI. Patients with a BMI < 25 kg/m^2^ were assigned to the nBMI group. The iBMI group consisted of HNC patients with a BMI ≥ 25 kg/m^2^. The classification of patients into the BMI-dependent groups was based on the WHO recommendations [88]. All patients were treated at the Prof. Franciszek Łukaszczyk Memorial Hospital’s Oncology Center in Bydgoszcz, Poland. Participants were included in the study at the time of referral for planning radiotherapy using positron emission tomography–computed tomography (PET/CT). Patients were subjected to a histopathological examination. The histopathological analysis indicated that the study group included patients with G1 squamous cell carcinoma, G2 squamous cell carcinoma, nonkeratinizing G2 squamous cell carcinoma, or G2 keratinizing squamous cell carcinoma.

The control group consisted of 23 healthy people with anthropometric parameters similar to those of the nBMI patient group. The criteria for exclusion from the control group included chronic or acute diseases, such as cancer, diabetes, obesity, autoimmune disorders, and cardiometabolic disorders.

A questionnaire survey (Supplementary Questionnaire S1) was administered to study participants. The questions included in the questionnaire concerned addictions and other factors predisposing to the occurrence of HNC. The consent to participate in this research was voluntary and had no effect on the course of treatment. The study was accepted by the Bioethics Committee of the Nicolaus Copernicus University in Toruń, functioning at the Collegium Medicum in Bydgoszcz, Poland (consent no. KB 221/2018).

### 4.2. Study Design

Blood samples were collected in the morning after overnight fasting, between 7:00 a.m. and 9:00 a.m., from the median cubital vein by qualified medical personnel from the Department of Nuclear Medicine, Oncology Centre at Prof. Franciszek Łukaszczyk Memorial Hospital in Bydgoszcz, Poland. Each blood sample was collected into a 6 mL polypropylene tube with a clot activator and a gel separator. The samples were immediately transported under a reduced temperature condition to the laboratory of the Department of Medical Biology and Biochemistry, Faculty of Medicine, Ludwik Rydygier Collegium Medicum in Bydgoszcz, Nicolaus Copernicus University in Toruń, Poland. Centrifugation (6000× *g* for 10 min at 4 °C) was performed to separate the blood serum from the blood clot. After centrifugation, the blood serum was aliquoted into Eppendorf tubes. Samples were stored at a temperature of −80 °C for further biochemical analysis.

### 4.3. Biochemical Analysis

Serum concentrations of omentin-1, adipsin, adiponectin, C-peptide, ghrelin, GIP, GLP-1, glucagon, insulin, leptin, PAI-1, resistin, and visfatin were determined with the use of commercially available research kits. The following kits were used accordingly: an enzyme-linked immunosorbent assay kit for human omentin-1 (BioVendor–Laboratorni medicina a.s., Brno, Czech Republic), Bio-Plex Pro^™^ human diabetes adipsin and adiponectin immunoassays (Bio-Rad Laboratories Inc., Hercules, CA, USA), Bio-Plex Pro^™^ human diabetes 10-plex immunoassay for C-peptide, ghrelin, GIP, GLP-1, glucagon, insulin, leptin, PAI-1, resistin, and visfatin (Bio-Rad Laboratories Inc., Hercules, CA, USA). All analyses were performed in accordance with the manufacturer’s instructions. The enzyme immune assay kits used in the study contained the reagents necessary for the analysis, such as standard concentration analytes and blank and control samples. The optical density for the omentin-1 research kit was tested with the BMG Labtech CLARIOstar multimode microplate reader (BMG LABTECH GmbH, Ortenberg, Germany). Research kits manufactured by Bio-Rad Laboratories Inc. use fluorescence measurement to determine the level of individual analytes. Fluorescence was measured on a Bio-Plex^®^ 200 system (Bio-Rad Laboratories Inc., Hercules, CA, USA). The obtained results were expressed as pg/mL, ng/mL, or μg/mL.

### 4.4. Statistical Analysis

The study belongs to the category of observational case-control studies. Statistical analysis was performed with the use of Statistica 13.3 (TIBCO Software Inc., Palo Alto, CA, USA). The Shapiro-Wilk test to assess the hypothesis of normal distribution, Levene’s test to calculate the homogeneity of variances, and Pearson’s correlation coefficient to evaluate the relationship between the measured parameters were used in this study. For analyzes that met the conditions of normal distribution, a Student’s *t*-test for independent samples was performed, and the results were presented as a mean ± standard error of the mean (SEM). If the results did not meet the criterion of normal distribution, a non-parametric Mann–Whitney U test was used and the data was presented as a median and interquartile range (IQR–Q1; Q3).

## 5. Conclusions

The obtained results indicate a disruption of the endocrine function of adipose tissue and impaired glucose metabolism in the course of HNC. It has been shown that these changes are significantly intensified in obese HNC patients. In conclusion, neoplastic disease disturbs the homeostasis of glucose metabolism and increases the level of pro-inflammatory adipokines. Obesity, which is not a typical risk factor for HNC, may aggravate the negative metabolic changes associated with carcinogenesis. Among the analyzed parameters, ghrelin, visfatin, and PAI-1 seem to be particularly involved in the pathomechanisms of HNC development and/or progression. The levels of these analytes have been significantly altered in the HNC patients compared to healthy people. Interestingly, further changes have been observed in the obese HNC patients. Other markers of interest include adipsin and glucagon, which increased in the HNC patients compared to the controls. The parameters listed above seem to be related to head and neck carcinogenesis. Undoubtedly, further studies in this area are required; however, the presented results indicate new, potentially promising research directions.

## Figures and Tables

**Table 1:** Anthropometric and clinical characteristics of patients with head and neck cancer (HNC groups) and healthy volunteers (control group). Each value represents the mean ± S.E.M.

Parameter	HNC				Control		*p* _nBMI vs. iBMI_	*p*_nBMI vs. Control_
	nBMI		iBMI					
	Mean	SEM	Mean	SEM	Mean	SEM		
*n* (female/male)	23 (10/13)	-	23 (9/14)	-	23 (11/12)	-	0.770785	0.773350
Age [yrs]	64.130	1.464	64.696	2.073	62.217	1.478	0.824801	0.362876
Body mass [kg]	61.957	1.787	90.957	2.556	62.652	1.524	<0.000001	0.768436
Height [cm]	169.130	1.464	170.957	1.172	168.304	1.202	0.335512	0.664882
BMI [kg/m^2^]	21.601	0.445	31.089	0.755	22.066	0.358	<0.000001	0.420516
Ex-smoker or current smoker (y/n)	11/12	-	12/11	-	5/18	-	0.774186	0.065521

Abbreviations used: BMI: body mass index; HNC: head and neck cancer; iBMI: increased BMI group; nBMI: normal BMI group; SEM: standard error of mean. *p* < 0.05 was considered as statistically significant.

**Table 2:** Biochemical parameters with a statistically normal distribution analyzed in head and neck cancer (HNC) patients with respect to their body mass index (BMI).

Parameter	HNC				*p* Value
	nBMI		iBMI		
	Mean	SEM	Mean	SEM	
Adiponectin [µg/mL]	58.947	3.581	36.182	2.429	0.000004
Leptin [pg/mL]	1941.866	129.804	4030.817	273.135	<0.000001
Insulin [pg/mL]	334.688	25.264	684.051	42.573	<0.000001

Abbreviations used: iBMI: increased BMI group; nBMI: normal BMI group; SEM: standard error of mean. *p* < 0.05 was considered as statistically significant.

**Table 3:** Biochemical parameters with a statistically non-parametric distribution analyzed in head and neck cancer (HNC) patients with respect to their body mass index (BMI).

Parameter	HNC				*p* Value
	nBMI		iBMI		
	Median	IQR (Q1; Q3)	Median	IQR (Q1; Q3)	
Ghrelin [pg/mL]	282.341	229.697; 377.075	169.900	141.490; 196.700	0.000036
Omentin-1 [ng/mL]	701.330	660.220; 779.030	456.000	399.300; 688.960	0.000048
Adipsin [ng/mL]	677.935	475.431; 916.300	686.080	634.510; 815.832	0.775185
Resistin [pg/mL]	6846.331	5265.468; 7981.305	9089.872	7939.536; 12,098.560	0.000033
Visfatin [pg/mL]	1394.150	1171.907; 1647.002	1939.650	1692.205; 2298.355	0.000007
Glucagon [pg/mL]	1557.810	1452.890; 1639.300	1643.660	1398.320; 1891.490	0.783612
C-peptide [pg/mL]	611.900	512.850; 706.740	700.550	603.880; 834.100	0.019874
GLP-1 [pg/mL]	257.500	213.360; 277.600	297.200	277.200; 308.020	0.011522
PAI-1 [pg/mL]	3544.930	3122.099; 3956.693	4476.310	3924.160; 4979.300	0.004597
GIP [pg/mL]	315.300	290.650; 400.900	333.400	299.800; 388.600	0.660384

Abbreviations used: GIP: glucose-dependent insulinotropic polypeptide; GLP-1: glucagon-like peptide-1; iBMI: increased BMI group; IQR: interquartile range; nBMI: normal BMI group; PAI-1: plasminogen activator inhibitor-1. *p* < 0.05 was considered as statistically significant.

**Table 4:** Biochemical parameters with a statistically normal distribution analyzed in head and neck cancer (HNC) patients with normal body mass index values (nBMI) and a control group.

Parameter	HNC nBMI		Control Group		*p* Value
	Mean	SEM	Mean	SEM	
Omentin-1 [ng/mL]	727.701	18.184	721.247	20.433	0.814575
Adipsin [ng/mL]	680.076	49.457	443.729	20.163	0.000063
Visfatin [pg/mL]	1412.926	69.342	1241.184	29.803	0.027802
GIP [pg/mL]	339.807	22.837	290.509	12.393	0.064363

Abbreviations used: GIP: glucose-dependent insulinotropic polypeptide; SEM: standard error of mean. *p* < 0.05 was considered as statistically significant.

**Table 5:** Biochemical parameters with a statistically non-parametric distribution analyzed in head and neck cancer (HNC) patients with normal body mass index values (nBMI) and a control group.

Parameter	HNC nBMI		Control		*p* Value
	Median	IQR (Q1; Q3)	Median	IQR (Q1; Q3)	
Ghrelin [pg/mL]	282.341	229.697; 377.075	343.510	295.610; 434.450	0.034942
Adiponectin [µg/mL]	53.984	43.884; 75.405	45.673	39.945; 64.519	0.118805
Leptin [pg/mL]	1899.600	1416.650; 2393.790	1715.204	1576.584; 2998.286	0.253290
Resistin [pg/mL]	6846.331	5265.468; 7981.305	6781.360	5736.944; 7695.312	0.741750
Glucagon [pg/mL]	1557.810	1452.890; 1639.300	1429.350	929.720; 1595.870	0.008941
Insulin [pg/mL]	297.780	232.930; 420.230	284.970	212.290; 500.420	0.792065
C-peptide [pg/mL]	611.900	512.850; 706.740	684.038	431.604; 852.606	0.613355
GLP-1 [pg/mL]	257.500	213.360; 277.600	245.682	155.502; 291.314	0.333723
PAI-1 [pg/mL]	3544.930	3122.099; 3956.693	2926.950	2462.028; 3436.926	0.000661

Abbreviations used: GLP-1: glucagon-like peptide-1; IQR: interquartile range; PAI-1: plasminogen activator inhibitor-1. *p* < 0.05 was considered as statistically significant.

## Data Availability

Data are available on request due to privacy/ethical restrictions.

## Supplementary Materials

The following supporting information can be downloaded at: https://www.mdpi.com/article/10.3390/ijms24043283/s1, Supplementary Questionnaire S1.
