# Supplementary material for: Concentration of Selected Adipokines and Factors Regulating Carbohydrate Metabolism in Patients with Head and Neck Cancer in Respect to Their Body Mass Index
Source: Int J Mol Sci. 2023 Feb 7;24(4):3283. doi: 10.3390/ijms24043283 (PMC9959515; doi:10.3390/ijms24043283)
Supplement: Supplementary file 1 [file ijms-24-03283-s001.zip › ijms-2174357-supplementary.pdf]

Supplementary Questionnaire S1 (English translated version)

ID: ……………… (filled in by the examiner)

QUESTIONNAIRE

Dear Madam/Sir, we kindly ask you to complete the following questionnaire, which is part of the study *"Redox homeostasis, endocrine activity of adipose tissue and the role of inflammation in the carcinogenesis of head and neck tumors"*. Participation in the study is voluntary and free of charge, and the results obtained will be used only for scientific purposes. The following questions should be answered by selecting one option (unless otherwise stated in the question).
Thank you for your time.

1. Sex:
   - ☐ Female
   - ☐ Male
2. Age: …………………………… [yrs]
3. Body mass: ……………………. [kg]
4. Height: ………………………... [cm]
5. Professional activity:
   - ☐ Pupil
   - ☐ Student
   - ☐ Manual worker
   - ☐ Office worker
   - ☐ Unemployed
   - ☐ Pensioner

---

6. Are you ex-smoker or current smoker?
   - ☐ Yes
   - ☐ No
7. Do you drink alcohol?
   - ☐ Yes (please specify how often):
     - o A few times a week
     - o Weekly
     - o Every two weeks
     - o Less often
   - ☐ No
8. Do you practice any sport/exercise?
   - ☐ Yes (please specify how often):
     - o Every day
     - o A few times a week
     - o Weekly
     - o Less often
   - ☐ No
9. How do you define your diet?
   - ☐ Sustainable
   - ☐ Rich in protein
   - ☐ Rich in fat
   - ☐ Vegetarian
   - ☐ Other:……………………………………
10. Do you take vitamin supplements?
    - ☐ Yes
    - ☐ No
